# Accelerometer Derived Activity Counts and Oxygen Consumption between Young and Older Individuals

**DOI:** 10.1155/2014/184693

**Published:** 2014-05-26

**Authors:** Lucas Whitcher, Charilaos Papadopoulos

**Affiliations:** ^1^Department of Applied Arts and Sciences, University of Montana-Missoula College, Missoula, MT 95812, USA; ^2^Department of Kinesiology, Pacific Lutheran University, Tacoma, WA 98447, USA

## Abstract

The purpose of this investigation was to compare accelerometer activity counts and oxygen consumption between young and elderly individuals. Sixteen young (21.3 ± 2.5 yrs) and sixteen elderly (66.6 ± 2.9 yrs) participants completed 30 minutes of resting oxygen consumption to determine resting metabolic rate and four 6 min walking intensities ranging from 27 to 94 m·min^−1^. Resting oxygen uptake was significantly lower for the older participants. Exercise oxygen consumption was significantly higher for the elderly group. There were no significant differences in activity counts between groups at each of the exercise intensities. When using measured resting metabolic rate, activity counts of 824 and 2207 counts·min^−1^ were associated with moderate (3 METs) physical activity intensity for the older and young participants, respectively. However, using standard resting metabolic rate (3.5 mL·kg^−1^·min^−1^), activity counts of 784 and 2009 counts·min^−1^ were associated with moderate physical activity intensity for the elderly and young participants, respectively. These findings indicate that activity counts are similar across age groups even though the oxygen consumption of exercise is greater among elderly individuals.

## 1. Introduction

Despite the importance of physical activity in maintaining and improving health and quality of life in those individuals over the age of 65, research suggests that less than 3% of elderly Americans [1] comply with the American College of Sports Medicine's physical activity recommendations [2, 3]. Given the correlation between physical activity and increased health, it is of interest to accurately measure physical activity levels within a population. However, the ability to quantify physical activity in individuals can be a problematic task. In addition, because the current recommendations for physical activity include both intensity and duration, there is a compulsory need that measurement tools do the same. Criterion methods, such as doubly labeled water and open-circuit spirometry, may not be plausible when examining large populations due to cost and/or an inability to obtain frequency and intensity of physical activity. Furthermore, the commonly used self-report instruments are subjective and rely on recall information, which may be limited when applied to an older population [4].

Accelerometers offer detailed information over extended periods pertaining to physical activity behavior. This information can be used to provide estimates of time spent in different levels of physical activity intensities and overall patterns of physical activity. Unfortunately, few applications to the elderly population have been made [5] and the raw output data can be difficult to interpret [1]. Moreover, accelerometer activity count cut-points used to determine physical activity intensity levels have been established from studies testing young participants [6–9]. To date, there are only very few studies that have sought to determine an activity count cut-point for determination of moderate physical activity intensity for elderly adults [8, 10, 11] and only one study has evaluated the relationship between oxygen consumption and activity counts of younger and older adults [8]. Furthermore, since investigations have shown that elderly individuals require a greater metabolic cost for walking at the same absolute intensity as young individuals [12, 13], it seems necessary to make such a comparison. Lastly, evidence suggests that the standard 3.5 mL·kg^−1^·min^−1^ may not represent the resting metabolic rate of the general population [14, 15] and that older adults may have a lower resting metabolic rate compared to younger individuals [14, 16]. Due to those differences at rest and during exercise between younger and older adults, the researchers hypothesized that a lower resting metabolic rate and higher oxygen consumption during exercise with similar accelerometer derived activity counts may result in a lower activity count threshold associated with moderate exercise intensity for older adults.

Therefore, the first purpose of this investigation was to compare accelerometer derived activity counts and oxygen consumption, measured during treadmill walking, between young and elderly participants. Secondly, we sought to determine the relationship between metabolic equivalent (MET) and accelerometer derived activity counts for each group of participants by using estimated and measured MET levels.

## 2. Methods

### 2.1. Participants

Thirty-two individuals, 16 elderly (age 66.6 ± 2.9 yrs) and 16 young (age 21.3 ± 2.5 yrs), recruited from the local community agreed to participate in this study. Participants were recruited from flyers posted at recreational facilities (e.g., senior centers) and bulletin boards at various locations (e.g., university, grocery stores). The primary inclusion criterion was the ability to walk unassisted on a treadmill. However, secondary inclusion criteria were used to further screen participants. Only participants that were healthy, free of injury within the past six months, and not taking medication (e.g., antidepressants, beta-blockers) or stimulants (e.g., caffeine, smoking) known to influence energy expenditure or exercise capacity were tested. All participants provided written informed consent and approval was obtained by the Central Washington University Human Subjects Review Committee.

### 2.2. Procedures

Participants attended a familiarization session prior to testing. During the familiarization session, participants rested for 10 minutes in a seated position while breathing through a mouthpiece and then walked at the various intensities to familiarize them with the walking speeds and grade on the treadmill. At least two days and no more than seven days after the familiarization session, a testing session was scheduled. Even though the majority (27 out of 32) of the tests were performed in the morning (before 9 a.m.), there were some tests that were performed at a later time mainly due to participant availability. Participants were instructed to refrain from caffeine and exercise on the day of testing. Also, participants were asked to withhold food consumption 3-4 hours prior to testing. Participants reported to the laboratory and after 10 min of rest to determine compliance with pretest instructions and answer last minute questions; height was recorded to the nearest centimeter (MED ART, St. Louis, MO) and weight was measured to the nearest 0.5 kg (Detecto-Medic, Brooklyn, NY). Body density and percent body fat were determined from skinfold measurements (Lange Skinfold Calipers, Cambridge Scientific Instruments Inc., Cambridge, MD) as described by Pollack et al. [17].

### 2.3. Resting Metabolic Rate

Indirect calorimetry (True Max 2400, Parvo Medics Inc., Sandy, Utah) was used for the measurement of resting metabolic rate. Gas analyzers were calibrated before each test with a gas tank of known concentration (16% O_2_, 4% CO_2_). Respiratory flows were measured with a pneumotachometer calibrated before each test with a syringe of known volume (3.0 L). Participants were then fitted with a mouthpiece that incorporates a two-way breathing valve apparatus (Hans Rudolph Inc., Kansas City, MO.). Initially participants were in a supine position but during pilot testing older participants indicated that they were uncomfortable and therefore all participants remained awake in a seated position for 30 min while expired gases were collected. Expired gases were sampled in 30 s intervals and averaged oxygen uptake values ($\dot{\text{V}}$O_2_), both relative (mL·kg^−1^·min^−1^) and absolute (L·min^−1^), obtained from minutes 15 to 25 were used to calculate an individual's resting metabolic rate. In addition, heart rate (Polar Electro, USA) was monitored throughout the entire resting period and averaged during minutes 15–25 and this value was considered as the individual's resting heart rate.

### 2.4. Treadmill Exercise

Similar to the procedures of Copeland and Esliger [10], the treadmill bout consisted of 6 min stages with 5 min seated rest periods between stages. The four treadmill stages included walking at speeds of 27, 54, and 80 m·min^−1^ (1.0, 2.0, and 3.0 mph, resp.) and 0% grade, performed in random order, and a final stage of 94 m·min^−1^ (3.5 mph) and 2% grade. These intensities were selected to induce light, moderate, and vigorous physical activity. Based on pilot testing, the 94 m·min^−1^ and 2% grade stage was excluded from the random selection because this intensity was too difficult for some individuals to begin with, particularly in the absence of a warm-up and could have resulted in higher oxygen consumption on subsequent stages. All trials were performed on the same Quinton Q65 treadmill (Quinton Instruments Company, Seattle, WA). The speed and the grade were checked before each trial. Oxygen consumption during the treadmill bout was calculated by averaging the 30 s sampling rate values obtained in the final 3 min of each treadmill stage completed. In order to determine measured MET value per stage, averaged oxygen consumption per stage was divided by the individual's resting metabolic rate (measured $\dot{\text{V}}$O_2_/measured resting metabolic rate) and by the standard (3.5 mL·kg^−1^·min^−1^) metabolic rate (measured $\dot{\text{V}}$O_2_/3.5).

### 2.5. Accelerometers

Prior to the treadmill bout, participants were fitted with two Actigraph (Pensacola, Florida) accelerometers (one GT1M model and one GT3X model). The GT1M (mass, 27 g; width, 38 mm; height, 37 mm; thickness, 18 mm) is a newer smaller version of the discontinued Actigraph model 7164. This updated model has demonstrated similar activity counts per minute, when compared with the Actigraph 7164, at treadmill speeds of 54, 80, and 107 m·min^−1^ [18]. Unlike the GT1M model, which senses vertical accelerations, the GT3X detects accelerations along three planes of motion. According to the manufacturer, the GT1M and the GT3X models utilize the exact same activity filtering algorithm. Both activity monitors were initialized before each trial using the same laptop computer and using the ActiLife low frequency extension. In addition, the activity monitors were marked so that the same units were used for each participant. Accelerometers were securely mounted side by side along the anterior-axillary line of the right hip with an adjustable nylon belt. The activity counts recorded by the GT1M and the vertical axis activity counts from the GT3X were summed using 1 min sampling intervals (epochs) and averaged for the final 3 min of each treadmill stage. For the purposes of this investigation, the average activity counts from the GT1M and GT3X were used in the statistical analysis.

### 2.6. Statistical Analysis

Body weight, height, and body composition were evaluated for significant differences between groups using a two-tailed independent* t*-test. Likewise, two-tailed independent* t*-tests were used to determine differences in heart rate and oxygen consumption between groups at rest. A two-way repeated measures (RM) analysis of variance (ANOVA) with one within factor (exercise intensity) and one between factor (elderly versus young) was used to evaluate differences between groups in averaged activity counts, oxygen consumption, and MET values for each of the intensities. To examine for differences between activity counts derived from the GT1M and GT3X Actigraph models, a two-way ANOVA was used as well. If statistical significance was found, Bonferroni* post hoc* test was used. A linear regression analysis was used to determine the relationship between measured METs and averaged vertical axis activity counts and between estimated METs and averaged vertical axis activity counts for each group (young and elderly). The prediction equation was determined from the entire data set for each group. Finally, activity counts corresponding to moderate intensity (3 METs) exercise were calculated from the developed regression equations. All data were analyzed using SPSS version 16.0. Data are expressed as means ± SD, and statistical significance was set at *P* < 0.05.

## 3. Results

Thirty-two individuals participated in the investigation (16 elderly and 16 young; Table 1). The young and elderly groups were similarly matched for gender (9 males and 7 females; 8 males and 8 females, resp.).

All participants completed the first three stages of treadmill walking. However, two of the elderly participants were unable to perform at the 94 m·min^−1^ and 2% grade. In one case, the participant declined to begin the final stage due to fatigue. In the other case, the participant began the final stage but requested to stop because of difficulty maintaining pace. Activity counts measured by the GT1M and GT3X Actigraph models are presented in Table 2.

There were no differences in activity counts between accelerometers for any of the exercise intensities, although the two-way RM ANOVA revealed significant difference for activity counts among the different intensities (Table 2). At rest, oxygen uptake relative to body mass among the elderly was significantly lower than the young group (3.49 ± 0.50 and 3.89 ± 0.28 mL·kg^−1^·min^−1^, resp.). There were no differences between groups in relative oxygen uptake (mL·kg^−1^·min^−1^) and heart rate at rest (Table 3). However, the two-way RM ANOVA showed a significant interaction between groups and exercise intensities. Bonferroni* post hoc *analysis indicated that oxygen uptake, measured MET, and heart rate values were significantly greater for the elderly participants at each of the intensities performed (Table 3).

**Figure 1:**
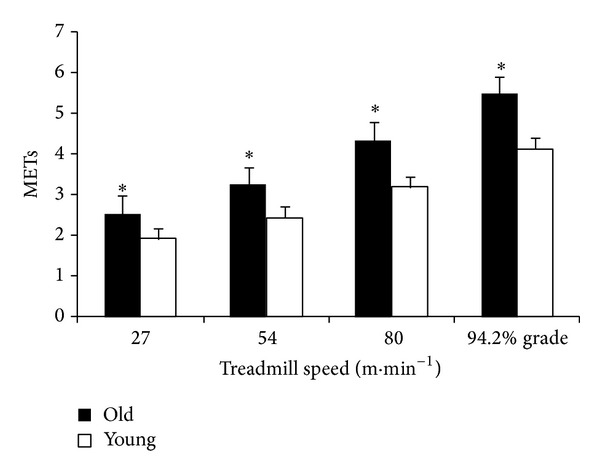
Treadmill speed and corresponding measured MET values among young and elderly participants. *Significantly greater than the younger group, *P* < 0.05.

Measured MET values were significantly greater among the elderly participants for each of the treadmill intensities (Figure 1).

**Figure 2:**
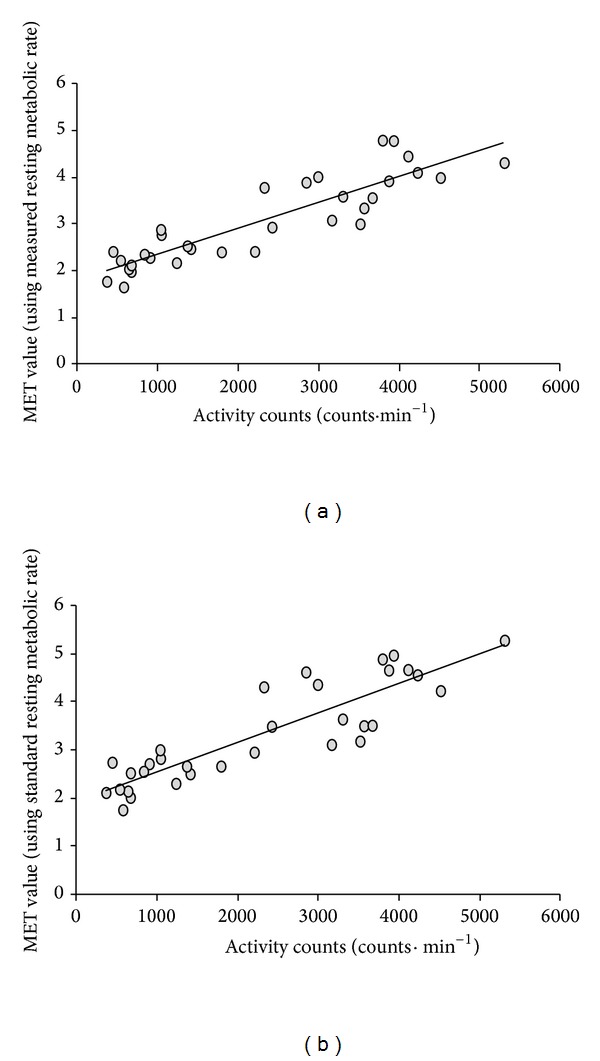
(a) Relationship between MET values and activity counts for young group using measured metabolic rate (*R* ^2^ = 0.80, standard error of estimate = 0.40). (b) Relationship between MET values and activity counts for young group using standard (3.5 mL·kg·min^−1^) metabolic rate (*R* ^2^ = 0.82, standard error of estimate = 0.41).

A strong correlation (*r* = 0.89) between averaged activity counts and measured MET values existed among the young group (Figure 2(a)). Activity counts and estimated MET values were also strongly correlated (*r* = 0.91) for the young group (Figure 2(b)).

**Figure 3:**
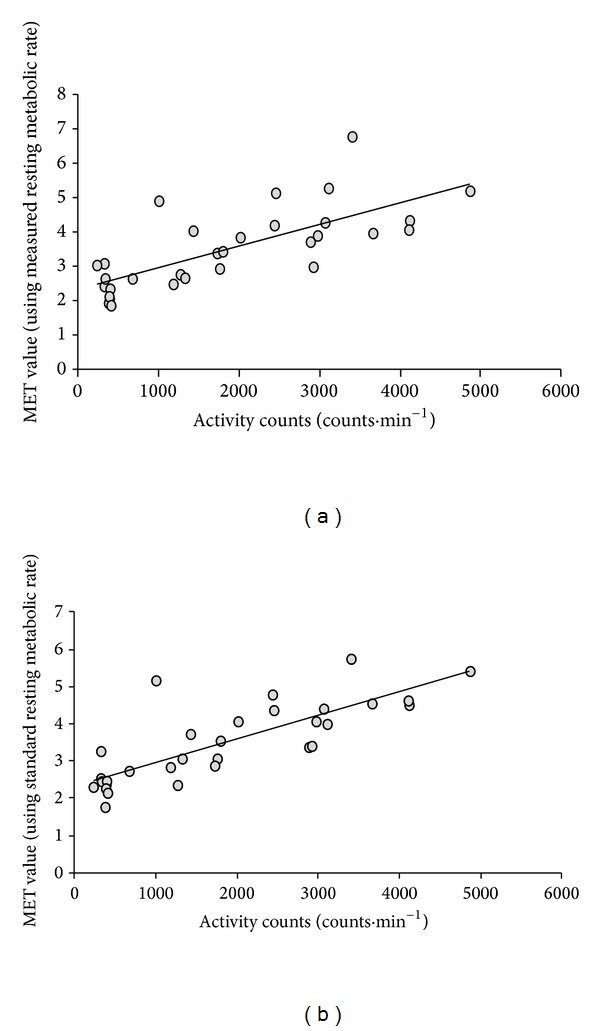
(a) Relationship between MET values and activity counts for elderly group using measured metabolic rate (*R* ^2^ = 0.57, standard error of estimate = 0.83). (b) Relationship between MET values and activity counts for elderly group using standard (3.5 mL·kg·min^−1^) metabolic rate (*R* ^2^ = 0.52, standard error of estimate = 0.85).

The relationships between activity counts and measured METs (Figure 3(a)) were moderate (*r* = 0.72) as it was the relationship (*r* = 0.76) between averaged activity counts and estimated METs (Figure 3(b)) for the older participants.

Based on the linear regression equations accelerometer activity counts of 824 (95% CI: 226–1719) and 2207 (95% CI: 1730–3008) counts·min^−1^ were associated with moderate (3 METs) physical activity intensity for the elderly and young participants, respectively, when using measured resting metabolic rate. Using the standard 3.5 mL·kg^−1^·min^−1^, linear regression equations indicated that moderate (3 METs) intensity physical activity was associated with 784 (95% CI: 104–1859) and 2009 (95% CI: 1267–2320) counts·min^−1^ for the elderly and young participants, respectively.

## 4. Discussion

The purpose of this investigation was to compare treadmill derived activity counts, oxygen consumption, and MET values between young and elderly participants. Despite the widespread use of accelerometers for obtaining objective measures of physical activity, few applications to older populations have been made. Consequently, this study provides valuable information pertaining to the plausibility of the use of accelerometers within an elderly population. The main finding of the study is that despite no significant differences in accelerometer derived activity counts between older and younger participants at various walking intensities, there were significant differences in relative oxygen consumption and MET values at rest and all exercise intensities indicating that moderate intensity physical activity for older adults may be determined at lower accelerometer derived activity counts than younger adults.

Oxygen uptake values obtained during treadmill exercise from the younger individuals in this study are in agreement with data reported in the literature. Leenders et al. [19] reported oxygen uptake values in a group of young adults (age 23.7 ± 4), who performed treadmill walking exercises at speeds of 54, 80, and 94 m·min^−1^, respectively. These values closely parallel those measured from the younger participants in our study (Table 3). Furthermore, oxygen consumption values associated with walking at the respective speeds in elderly individuals in this study are consistent with findings from previous investigations [10, 13].

In this investigation, elderly participants had significantly higher oxygen uptake values than the younger participants during treadmill walking across the range of intensities. Similar findings have demonstrated a 12% and 22% increase, among 65- and 80-year-old participants, respectively, in the energy cost of walking at speeds of 80 and 94 m·min^−1^, compared with 25-year-olds [13]. Energy expenditure per mile walked, among elderly, has been shown to be 21% greater than younger individuals [12]. Others have found 31% greater energy cost of walking in older (age 74 ± 3) compared to younger adults (age 27 ± 3) [20]. The average oxygen consumption demonstrated by the elderly participants in this investigation ranged from 14 to 19% greater than the younger participants across the selected speeds. The data in our study support the idea of a greater energy cost in the elderly when compared to a younger cohort for varying walking intensities. Nonetheless, the underlying reasons for the increased energy cost among elderly individuals are not fully understood.

Interestingly, activity counts (counts·min^−1^) did not differ between groups at any of the treadmill intensities performed. This is in agreement with the results by Miller et al. [8]. Several other studies have reported Actigraph activity counts per minute similar to ours for equivalent treadmill walking speeds [19, 21–23], although these studies have been specific to younger populations. Unfortunately, data concerning Actigraph activity counts produced during treadmill walking in older populations is limited. Therefore, few comparisons to this study can be made. However, an investigation similar to ours reported mean activity counts of 515, 1041, and 2504 counts·min^−1^ at speeds of 33.5, 54, and 80 m·min^−1^, respectively, in a group of elderly participants (age 69.7 ± 3.5) [10]. Although these values closely resemble ours, the activity counts obtained from our sample of elderly individuals tend to be slightly higher. This difference can be attributed to different models of Actigraph accelerometer used to determine activity counts. Copeland and Esliger [10] used the 7164 model whereas in the present study we used the GT1M and GT3X models. A study by Kozey et al. [15] has shown that activity counts from the GT1M model are slightly but significantly higher than the 7164 model.

Research has indicated that the standard 3.5 mL·kg^−1^·min^−1^ may not represent the resting metabolic rate of the general population and that resting metabolic rate may be lower in older individuals. Since previous studies by Copeland and Esliger [10] and Miller et al. [8] have not measured resting metabolic rate in older adults, this study aimed to determine measured MET values for each of the exercise intensities. To our knowledge, this attempt to identify the relationship between measured MET values and activity counts for a sample of elderly participants had not previously been performed. In agreement with previous research [14, 16], resting metabolic rate in our group of elderly individuals was significantly less than the younger participants. However, the average resting metabolic rate for the older participants was similar to the standard MET value of 3.5 mL·kg^−1^·min^−1^ whereas the average resting metabolic rate for the younger adults was significantly higher than the standard MET value of 3.5 mL·kg^−1^·min^−1^. The average resting metabolic rates for both older and younger participants in this study are higher than other studies [14–16]. Some research indicates that casual seated resting metabolic rate approximates 3.6 mL·kg^−1^·min^−1^ [24]. It is possible that the differences between the present study and previous studies are due to different positions during resting periods. While the mean measured MET value among the older individuals approached but was not equivalent to 6 METs at a walking speed of 94 m·min^−1^ 2% grade, five of them obtained values greater than 6 METs. At the intermediate treadmill speeds of 54 and 80 m·min^−1^, all of the elderly participants were exercising at or above a moderate intensity. In contrast, the younger participants did not reach a level of moderate physical activity intensity until a treadmill speed of 80 m·min^−1^. Furthermore, even at the fastest speeds, younger individuals were exercising well below a vigorous intensity.

Translating accelerometer activity counts to determine intensity of physical activity has proven to be a difficult task and has been questioned by some studies [25, 26]. The aim of the present study was not to develop any new regression equation. However, based on the data, we were able to determine an activity count threshold that is associated with moderate (3 METs) physical activity for this group of younger and older adults and compare this activity count threshold with current literature. The commonly used Actigraph activity count threshold for moderate physical activity intensity (≥1952 counts·min^−1^), developed by Freedson et al. [6], was derived from exercise tests involving young adults. Similarly, Actigraph activity count cut-points for moderate physical activity intensity ranging from 1810 to 2260 counts·min^−1^ have been shown in other studies testing adults aged 20–45. In agreement with previous studies of younger adults, the present study found activity count cut-points for moderate physical activity intensity to be 2207 and 2009 counts·min^−1^ depending on either measured or standard resting metabolic rate. In contrast to those studies, Copeland and Esliger [10] and Miller et al. [8] have indicated a moderate intensity threshold value of 1041 and 1566 counts·min^−1^, respectively, for their group of elderly individuals. In this investigation, the threshold value, equivalent to 3 METs, was 824 counts·min^−1^ when using measured resting metabolic rate and 784 counts·min^−1^ when using the standard resting metabolic rate. The activity count threshold associated with moderate intensity activity reported in this study is lower than the ones reported by previous research. Copeland and Esliger [10] did not attempt to determine cut-points of various intensities. The researchers simply used the activity counts that corresponded to a mean oxygen consumption of 13 mL·kg^−1^·min^−1^. In addition, Miller et al. [8] used a different methodology than previous research. Previous studies, including the present study, have used a discontinuous protocol. Participants exercised at various intensities for five minutes and rested between intensities for five or six minutes. Miller et al. [8] used a continuous walking and/or running protocol. It is therefore difficult to compare the activity counts reported in the present study and the one by Miller et al. [8].

The results of the present study, along with the results of previous research, may represent the need to establish agreeable metabolic equivalents for respective age groups to effectively determine physical activity intensity level activity count cut-points. These findings are of significant importance because recent epidemiological investigation pertaining to physical activity behaviors among elderly United States citizens reported a substantially low amount of time spent in moderate intensity activity. However, the activity count threshold employed for identifying moderate physical activity in adults aged 18–60^+^ was 2020 counts·min^−1^ [1]. It is possible that previous epidemiological reports may be greatly underestimating the amount of time elderly populations spend performing moderate physical activity.

There are some limitations in this study. This sample size of younger and older adults is small and therefore the activity count threshold that is associated with moderate (3 METs) physical activity for this group of participants might not be generalizable to all older and younger adults. In addition, there were no participants between the ages of 30 and 55. Troiano et al. [1] have shown that accelerometry counts per minute do not change between ages of 18 and 59. In addition, the activity count thresholds established by Freedson et al. [6] were based on similar age group as ours. Therefore, the purpose of this study was to examine the differences in oxygen consumption and activity counts between a group of younger individuals (similar to the one by Freedson et al. [6]) and a group of older individuals above 60 years. The majority of the participants exercised below vigorous intensity (<6 METs). This limits the study from determining an activity count threshold associated with vigorous intensity. The intensity of 94 m·min^−1^ and 2% grade was used to elicit vigorous intensity. Two of the older participants were unable to complete this stage. It is difficult to elicit vigorous intensity for older adults due to either their lack of fitness, fear, or functional limitations.

## 5. Conclusions

Despite similarity in accelerometer activity counts produced during treadmill walking across a range of treadmill speeds among elderly and young participants, an increased oxygen uptake among elderly participants was evident. Therefore, accelerometers appear to be valid in their detection of exercise intensity across age groups. Moreover, translating activity counts into categorical levels of physical activity intensity cannot be done in the same manner for young and elderly individuals. Measurement of resting metabolic rate may offer the most effective way for determining the relationship between activity counts and the intensity level of physical activity performed. Further research is necessary in order to better understand the relationship between accelerometer activity counts and physical activity intensity levels for an elderly population.

## Figures and Tables

**Table 1:** Participants characteristics.

	*n *	Age (yrs)	Weight (kg)	Height (cm)	Percent body fat
Young	16	21.3 ± 2.5	72.8 ± 12.3	173.1 ± 8.4	18.9 ± 11.4
Elderly	16	66.6 ± 2.9*	77.7 ± 18.5	166.5 ± 9.2*	24.4 ± 9.1

*Significantly different from the young group, *P* < 0.05.

**Table 2:** Activity counts (counts·min^−1^) during treadmill walking from uniaxial (GT1M) and triaxial (GT3X) accelerometers (*n* = 32).

	Treadmill speed (m·min^−1^)			
	27	54	80	94, 2% grade
Activity counts (GT1M)	302 ± 264^#^	1286 ± 481^#^	3000 ± 789^#^	3711 ± 881^#^
Activity counts (GT3X)	307 ± 276^#^	1184 ± 491^#^	2903 ± 817^#^	3651 ± 884^#^
Average activity counts	304 ± 271	1226 ± 483	2955 ± 809	3686 ± 882

^#^Significantly different among speeds, *P* < 0.05.

**Table 3:** Relative oxygen uptake (mL·kg·min^−1^), heart rate, averaged activity counts, and measured MET values at rest and during treadmill walking in young (*n* = 16) and elderly subjects (*n* = 14).

		Rest	Treadmill speed (m·min^−1^)			
			27	54	80	94, 2% grade
Young	$\dot{\text{V}}$O_2_	3.9 ± 0.3	7.6 ± 0.6^#^	9.4 ± 0.6^#^	12.4 ± 0.9^#^	16.0 ± 1.3^#^
	HR	67.3 ± 6.9	78.9 ± 7.7^#^	84.6 ± 6.7^#^	93.3 ± 8.3^#^	107.4 ± 13.9^#^
	MET		1.9 ± 0.2^#^	2.4 ± 0.3^#^	3.2 ± 0.3^#^	4.14 ± 0.4^#^
	AC		209 ± 130^#^	1188 ± 444^#^	2998 ± 739^#^	3689 ± 803^#^

Elderly	$\dot{\text{V}}$O_2_	3.5 ± 0.5*	8.6 ± 1.4^∗#^	11.0 ± 1.7^∗#^	14.7 ± 2.0^∗#^	18.5 ± 2.2^∗#^
	HR	69.8 ± 9.8	86.7 ± 10.9^∗#^	93.8 ± 10.5^∗#^	109.2 ± 15.4^∗#^	119.9 ± 14.6^∗#^
	MET		2.5 ± 0.4^∗#^	3.3 ± 0.6^∗#^	4.3 ± 0.7^∗#^	5.5 ± 0.9^#^
	AC		412 ± 352^#^	1267 ± 532^#^	2805 ± 891^#^	3506 ± 1032^#^

$\dot{\text{V}}$O_2_: relative oxygen consumption (mL·kg·min^−1^); HR: heart rate (bts·min^−1^); AC: averaged activity counts (counts·min^−1^).

*Significantly different from young group, *P* < 0.05.

^#^Significantly different among speeds, *P* < 0.05.
